# COVID-19 plasma exosomes promote proinflammatory immune responses in peripheral blood mononuclear cells

**DOI:** 10.1038/s41598-022-26457-8

**Published:** 2022-12-16

**Authors:** Lechuang Chen, Rui Chen, Min Yao, Zhimin Feng, Guoxiang Yuan, Fengchun Ye, Kien Nguyen, Jonathan Karn, Grace A. McComsey, Thomas M. McIntyre, Ge Jin

**Affiliations:** 1 Rammelkamp Center for Research and Department of Medicine, Case Western Reserve University School of Medicine, the MetroHealth System Cleveland, Cleveland, OH 44109 USA; 2 Department of Cardiovascular and Metabolic Sciences, Cleveland Clinic Lerner Research Institute, Cleveland, OH 44195 USA; 3 Department of Radiation Oncology, Penn State Cancer Institute, The Pennsylvania State University College of Medicine, Hershey, PA 17033 USA; 4 Department of Molecular Biology and Microbiology, Case Western Reserve University School of Medicine, Cleveland, OH 44106 USA; 5 Department of Pediatrics and Medicine, Case Western Reserve University School of Medicine, University Hospitals of Cleveland, Cleveland, OH 44106 USA

**Keywords:** Virology, SARS-CoV-2, Microbiology, Diseases, Infectious diseases

## Abstract

Elevated serum cytokine production in COVID-19 patients is associated with disease progression and severity. However, the stimuli that initiate cytokine production in patients remain to be fully revealed. Virus-infected cells release virus-associated exosomes, extracellular vesicles of endocytic origin, into the blood to deliver viral cargoes able to regulate immune responses. Here, we report that plasma exosomes of COVID-19 patients contain SARS-CoV-2 double stranded RNA (dsRNA) and stimulate robust production of interleukin-6 (IL-6), IL-8, tumor necrosis factor-α (TNF-α), and other inflammatory cytokines and chemokines by human peripheral mononuclear cells. Exosome depletion abolished these stimulated responses. COVID-19 plasma exosomes induced proinflammatory responses in CD4^+^ T cells, CD8^+^ T cells, and CD14^+^ monocytes but not significantly in regulatory T cells, Th17 T cells, or central memory T cells. COVID-19 plasma exosomes protect the SARS-CoV-2 dsRNA cargo from RNase and deliver the dsRNA into recipient cells. These exosomes significantly increase expression of endosomal toll-like receptor 3 (TLR3), TLR7, TLR8, and TLR9 in peripheral T cells and monocytes. A pharmacological inhibitor of TLR3 considerably reduced cytokine and chemokine production by CD4^+^ and CD8^+^ T cells but not by CD14^+^ monocytes, highlighting divergent signaling pathways of immune cells in response to COVID-19 plasma exosomes. Our results identify a novel model of intercellular crosstalk following SARS-CoV-2 infection that evoke immune responses positioned to contribute to elevated cytokine production associated with COVID-19 progression, severity, and long-haul symptoms.

SARS-CoV-2 causes COVID-19, which has spread across the globe, precipitating a current global health crisis, with over 5 million deaths worldwide since its first detection in December 2019^1–3^. COVID-19-related mortality is primarily caused by acute respiratory distress syndrome (ARDS); a cytokine storm is considered the main cause of ARDS^4^. During a cytokine storm, blood circulating immune cells, including T lymphocytes and monocytes, are recruited into the lung, producing unnecessarily large amounts of inflammatory cytokines and chemokines^5–8^. These agents include interleukin-6 (IL-6), IL-2, IL-7, granulocyte-colony stimulating factor (G-CSF), monocyte chemoattractant protein-1 (MCP-1), tumor necrosis factor-α (TNF-α), and many other cytokines and chemokines^9–11^. Overexpression of these cytokines can lead to significant tissue damage and lung injury^12^. Moreover, multiorgan damage and injury are more commonly observed in patients with a more severe form of the disease^13^.

SARS-CoV-2 is a positive sense single-stranded RNA enveloped virus with a genome of nearly 30,000 nucleotides^14^. Viral infection primarily reflects binding between its membrane glycoprotein spike and the angiotensin-converting enzyme 2 (ACE2) receptor on select host human cell surfaces^15^. Once inside the host cell, virus-infected cells can release virus-associated extracellular vesicles (EVs)^16–18^. EVs are lipid bilayer-enclosed structures secreted under physiological and pathological conditions by most types of cells that contain proteins, nucleic acids, metabolites, and lipids^19^. Based on biogenesis and sizes, EVs are classified into three main groups: exosomes (derived from endosomal multivesicular bodies, 20–150 nm in diameter), microvesicles (budding from the existing plasma membrane, 150–1000 nm in diameter), and apoptotic bodies (released by cells undergoing programmed cell death, 1–5 μm in diameter)^12,20,21^. Among these EVs, exosomes and microvesicles transfer their associated protein and ribonucleic acid cargoes to recipient cells as efficient mediators of cell-to-cell communication^20,22^. It has been proposed that SARS-CoV-2 may be directed into the exosomal pathway with its RNA and proteins, including the spike protein, packaged into exosomes destined for secretion^23^. These exosomes can interact with immune cells, such as T cells, macrophages, and dendritic cells, to modulate proinflammatory immune responses.

Exosomes play pivotal roles in spreading and increasing the adverse effects of viruses; however, knowledge of SARS-CoV-2-associated plasma exosomes in patients and the host response remains limited. Exosomes from the plasma of COVID-19 patients harbor tenascin-C and fibrinogen-β, which are responsible for triggering inflammatory signals in distant cells^24^. Plasma exosomes from COVID-19 patients contain viral RNA in the exosome cargo identified by using reverse transcription-droplet digital polymerase chain reaction (RT-ddPCR) but not viral proteins as determined by untargeted proteomic analysis^25^. However, Pesce et al. reported the presence of SARS-CoV-2 S protein in plasma exosomes recovered from COVID-19 patients using anti-tetraspanin immunoprecipitated exosomes and exosome detection chips^26^. Coronaviruses, including SARS-CoV-2, produce double-stranded RNA (dsRNA) intermediates early on in the infection cycle^27^. Host cells may pack viral dsRNA into exosomes to regulate innate immune responses and the pathogenesis of nonpermissive cells^17,18,28^.

In this study, we investigated the effect exosomes derived from COVID-19 patients have on the immune response of circulating leukocytes. We purified and characterized exosomes from plasma specimens from hospitalized COVID-19 patients at the time of admission and from those same patients later in their hospitalization at the University Hospitals Cleveland Medical Center, Cleveland, Ohio. We identified SARS-CoV-2 dsRNA sequences in COVID-19 plasma exosomes, which stimulated the production of IL-6, IL-8, TNF-α and other cytokines by PBMC from healthy donors. However, exosome-depleted COVID-19 plasma failed to effect cytokine expression by PBMCs, suggesting a potential role of SARS-CoV-2-associated exosomes to contribute to the elevation of serum cytokine levels in COVID-19. A specific dsRNA/TLR3 inhibitor blocked the expression of cytokines by T lymphocytes, but not by monocytes, in response to COVID-19 plasma exosomes. In addition, COVID-19 plasma exosomes stimulated expression of TLR3, TLR7, TLR8, and TLR9 by PBMC. Our findings indicate a novel role of crosstalk between SARS-CoV-2 infection and immune responses through plasma exosomes in stimulating peripheral blood immune cell cytokine production. SARS-CoV-2-associated exosomes may participate in the aberrant function of other cell types and organs that lack the ACE2 high affinity receptor for SARS-CoV-2 in patients.

## Results

### Plasma exosomes from COVID-19 patients promote cytokine production in peripheral blood mononuclear cells (PBMCs)

We obtained plasma specimens (200–250 µl each) from 50 hospitalized COVID-19 patients early upon admission and again later in their hospitalization from the COVID-19 and Coronavirus Biorepository at the University Hospitals Cleveland Medical Center (UHCMC), Cleveland, OH (Supplementary Table S1). We also received plasma specimens from age- and gender-matched hospitalized non-COVID-19 (non-COVID) donors from the UHCMC as controls. These individuals were age 15 or higher. Among 10 patients who had available laboratory tests for IL-6, nine presented higher IL-6 serum levels (8.3–255.8 pg ml^−1^) during hospitalization than reference individuals (≤ 2 pg ml^−1^). Among all patients, 92% had high C reactive protein levels during hospitalization (Supplementary Table S1). We purified plasma exosomes using a differential ultracentrifugation protocol and quantified plasma exosome yields based on acetylcholinesterase activity^17,18^. Exosome-depleted plasma from the same patients/donors was generated simultaneously and used as autologous controls. The yield of plasma exosomes was typically approximately 2 × 10^9^ particles from approximately 200 µl of each plasma sample. Given the similarity in size and density between SARS-CoV-2 virions and exosomes, COVID-19 plasma exosomes may contain virions due to cosedimentation of exosomes and viral particles. To eliminate viral interference in potential functionalities of plasma exosomes, we heated the plasma samples from COVID-19 patients and non-COVID donors at 57 °C for 30 min to inactivate the virus^14^, a procedure that does not affect the stability of plasma exosomes before exosome preparation^29,30^. Plasma exosomes and exosomes isolated from culture supernatants of Jurkat T cells exhibited the same size distribution as determined by nanoparticle tracking analysis using ZetaView (Supplementary Fig. S1a).

To determine whether plasma exosomes from COVID-19 patients contained SARS-CoV-2 components, we used a one-step RT-PCR platform suitable for qualitative detection of SARS-CoV-2 nucleic acids in saliva and the nasal swab samples without the need for RNA extraction (RayBiotech Inc.). We detected exosomal SARS-CoV-2 nucleic acids in over 90% of COVID-19 patients (45 positives vs. 5 negatives) following the manufacturer’s protocol. Among 50 plasma exosome samples derived from patients early in hospital admission, 41 (82%) were SARS-CoV-2 positive. However, only 10 out of 50 plasma exosome samples (one sample had no data) from the same patients later in their hospitalization were SARS-CoV-2 positive, suggesting diminishing release of exosomes from infected cells during hospitalization. To quantify SARS-CoV-2 nucleic acid sequences in COVID-19 plasma exosomes, we extracted total RNA from COVID-19 plasma exosome samples of 8 patients for reverse transcription and subsequent quantitative PCR (RT-qPCR) using primer sets that detect SARS-CoV-2 S and N genes (ScienCell, Inc.). We detected SARS-CoV-2 RNA, either envelope spike protein 1 (S) RNA or nuclear (N) gene RNA or both, in 7 out of 8 COVID-19 plasma exosome samples. Specifically, S RNA was identified in 5 exosome samples, while N RNA was identified in 3 samples from patients early in the admission. In those same patients who were in their later hospitalization.

However, S and N RNA was detected in 3 and 2 plasma exosome samples, respectively (Table 1). Most importantly, we detected viral RNA in COVID-19 plasma exosomes in some patients up to 86 days after infection (Pt0001, 86 days; Pt0959, 8 days; Pt2077, 9 days). Our data indicate the presence of SARS-CoV-2-associated sequences in exosomes in the circulation of patients during the acute phase of COVID-19 and, importantly, the potential for persistence of these sequences in COVID-19 patient plasma exosomes approximately 3 months after recovery. To determine whether the detected viral nucleotide acids were derived from SARS-CoV-2-infected cells, and not contaminating virions, we transfected SARS-CoV-2-ΔN/EGFP BAC or SARS-CoV-2-ΔN/EGFP-alpha BAC, in which the viral N gene was replaced by the green fluorescent protein (GFP) cDNA in the viral genome, into Vero E6 cells for exosome isolation. We found that exosomes isolated from culture supernatants of SARS-CoV-2-ΔN/EGFP VERO E6 cells contained the viral S gene using RT-qPCR, but not the nuclear N gene (Table 2).

**Table 1:** Viral RNA in COVID-19 plasms exosomes.

Exosome samples	S gene (E/L)	N gene (E/L)	Days (E/L)
Pt0001E/L	3.5/–	23/36.9	86
Pt0947E/L	21.1/–	8.6/–	23
Pt0959E/L	37/22.7	37.1/33	8
Pt1830E/L	–/–	–/–	16
Pt1846E/L	–/30.1	–/–	5
Pt1901E/L	16.8/30.4	–/–	5
Pt1963E/L	–/37.5	–/–	5
Pt2077E/L	17.7/24.8	–/–	9
NON-COV2	–	–	
NON-COV3	–	–	
NON-COV4	–	–	
pos ctrl1^a^	8.7	11.5	
pos ctrl2^a^	17.15	14.2	

Pt0001E/L–Pt1963E/L, COVID-19 patients earlier (E) and later (L) in their hospitalization; (E/L), viral RNA RT-qPCR in plasma exosomes from COVID-19 patients earlier (E) and later (L) in their hospitalization, respectively. Cycles of qPCR presented; NON-COV, non-COVID samples; ^a^positive control RNA (pos ctrl) from the manufacture; –, not detected by RT-qPCR.

**Table 2:** Viral RNA in exosomes from culture supernatants of cell lines.

Exo samples	S gene	N gene	*ATCB*
SARS-COV-2	16.2	–	21.4
SARS-COV-2 alpha	19.2	–	21.9
A549 N&S	22.8	23.9	35.3
pos ctrl^a^	16.5	31.7	21.0

^a^Positive control (pos ctrl) RNA provided by the manufacture; –, not detected by qPCR; *ATCB*, human β-actin gene.

Exosomes from culture media of A549 cells that overexpressed both S and N genes, as a positive control, were positive for both S and N genes. Our results are consistent with a recent report identifying the presence of SARS-CoV-2 RNA in exosomal cargo^25^ and suggest that the plasma from COVID-19 patients contains exosomes from SARS-CoV-2 infected cells.

### COVID-19 plasma exosomes stimulate immune responses in PBMC

The production of cytokines, particularly IL-6, IL-8, and TNF-α, is associated with the progression and severity of COVID-19^31,32^. To determine the response of immune cells to plasma exosomes, we first treated PBMC isolated from healthy donors with exosomes isolated from the plasma of COVID-19 patients, exosomes isolated from non-COVID plasma, and lipopolysaccharides (LPS) for 16 h, followed by flow cytometry to determine the abundance of intracellular IL-6, IL-8, and TNF-α in PBMC gated for CD3^+^ lymphocytes. We found that COVID-19 plasma exosomes and LPS stimulated significant production of IL-6, IL-8, and TNF-α in CD3^+^ lymphocytes compared with those treated with non-COVID plasma exosomes, establishing that PBMC responded to COVID-19 plasma exosomes (Supplementary Fig. 1b,c).

**Figure 1:**
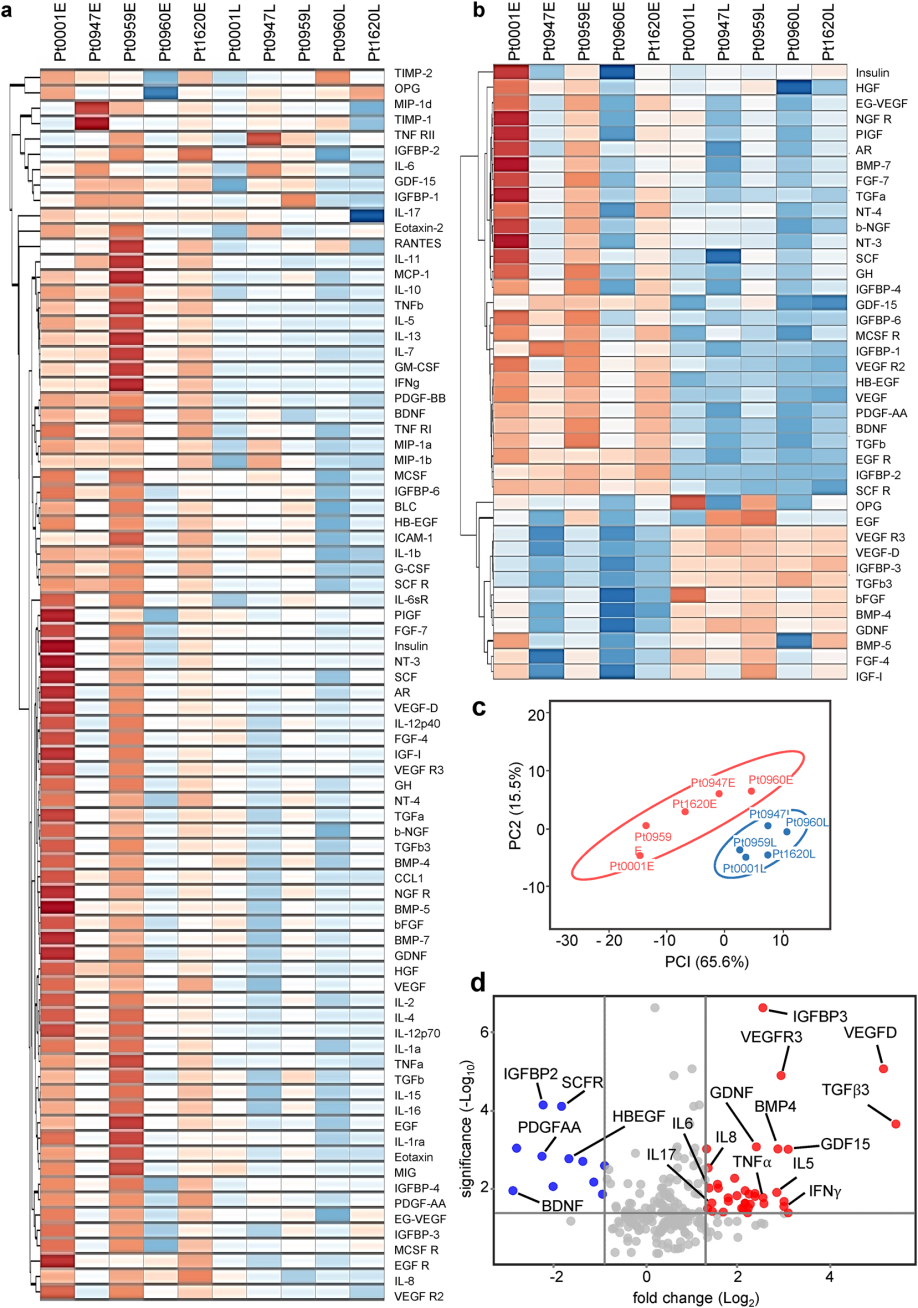
Profiles of cytokine production in PBMCs treated with COVID-19 plasma exosomes. Heatmap of inflammatory cytokine antibody arrays (**a**, G7 slide; **b**, G8 slide, RayBiotech Inc.) using culture supernatants from PBMC treated with COVID-19 plasma exosomes derived from patients early in their admission (Pt0001E to Pt1620E) and the same patients later in their hospitalization (Pt0001L to Pt1620L). The average age of the patients was 70.4. PBMC were treated with exosomes (4 × 10^9^ ml^−1^) for 16 h at 37 °C in RPMI media. (**c**) Principal components analysis plot of antibody array results. (**d**) Volcano plot of significantly expressed cytokines and chemokines based on the antibody arrays.

To determine whether SARS-CoV-2-associated exosomes played a role in the regulation of immune responses, we treated PBMC with exosomes (4 × 10^9^ ml^−1^, equivalent to exosomes purified from 200 to 300 µl of plasma) from plasma samples collected from five patients representing a wide span of hospital lengths of stay (8–86 days), followed by semiquantitative antibody arrays to measure cytokines released by PBMC. Non-COVID plasma exosomes were used as controls to normalize cytokine production. We found that plasma exosomes from COVID-19 patients upon admission (E) significantly stimulated the production of cytokines and chemokines, including IL-6, IL-8, and TNF-α, compared with those from the same patients later in their hospitalization (L, Fig. 1a,b). This proves that SARS-CoV-2-associated plasma exosomes have the potential to contribute to the elevation of cytokines observed in COVID-19 patients^31^. The response of PBMC to COVID-19 plasma exosomes from patients early and later in their hospitalization was clearly separated in the principal components analysis (PCA) space (Fig. 1c), highlighting a difference in the cargos of plasma exosomes over the course of COVID-19. We quantified the change in proteins released from PBMCs in response to COVID-19 plasma exosomes based on their statistical significance using a simple linear model. Our analyses revealed a dramatic increase in IL-6, IL-8, TNF-α, vascular endothelial growth factor D (VEGFD), vascular endothelial growth receptor 3 (VEGFR3), transforming growth factor-β 3 (TGFβ3), IL-5, insulin like growth factor binding protein 3 (IGFBP3), granulocyte–macrophage colony-stimulating factor (GM-CSF), growth differentiation factor 15 (GDF15), and IFNγ, with brain derived neurotrophic factor (BDNF), mast/stem cell growth factor receptor Kit (SCFR), heparin binding-epidermal growth factor receptor (HBEGF), platelet-derived growth factor alpha polypeptide a (PDGF-AA), and IGFBP2 proteins compared with all cytokines relative to their fold changes in volcano plots (Fig. 1d). Our results suggest that COVID-19 plasma exosomes have the potential to contribute to the immune response of PBMC.

**Figure 2:**
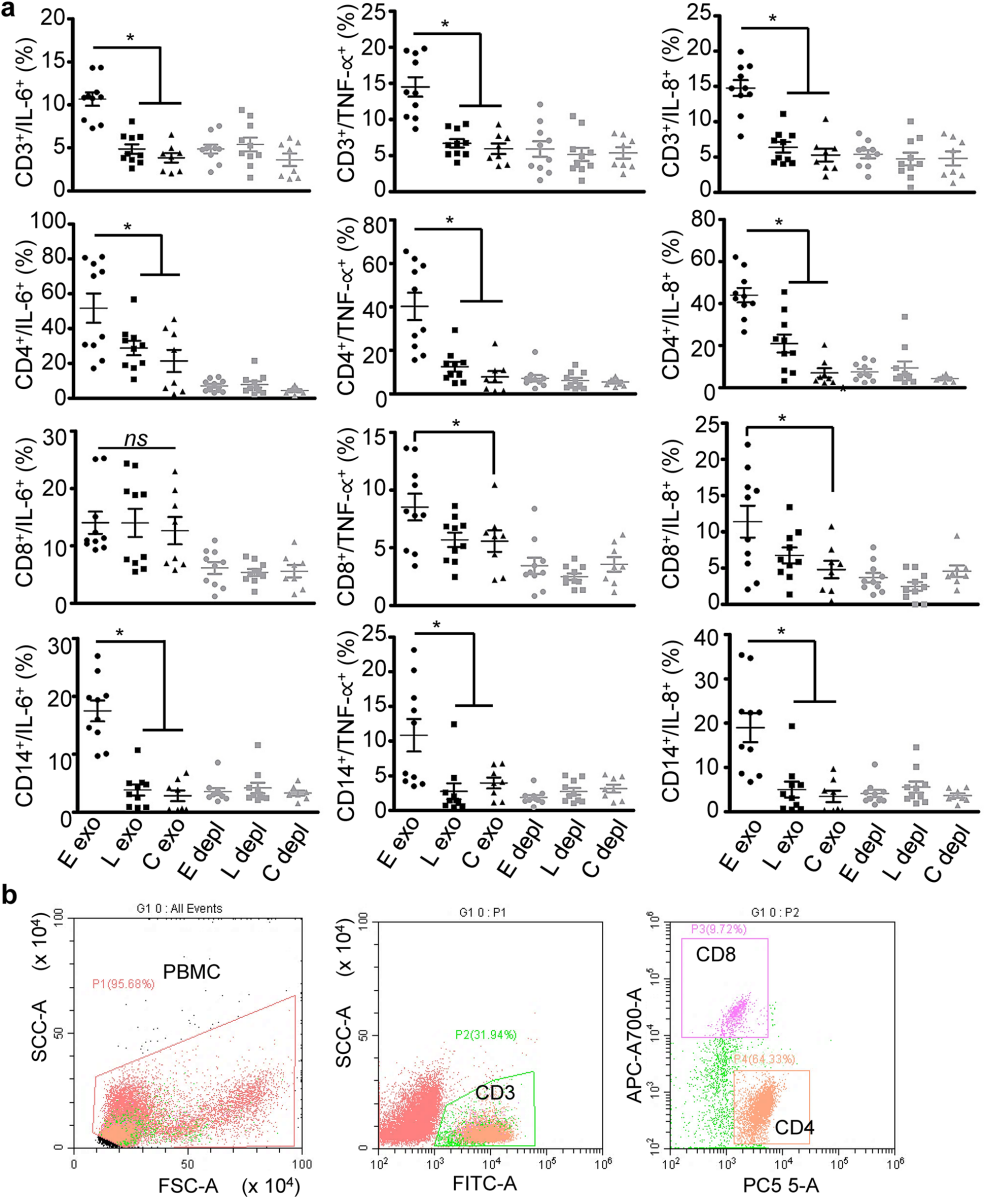
Cytokine production of PBMC treated with plasma exosomes. (**a**) Expression of IL-6, IL-8, and TNF-α in PBMC gated on CD3^+^, CD4^+^, CD8^+^, and CD14^+^. PBMCs were treated with plasma exosomes (4 × 10^9^ ml^−1^) from COVID-19 patients upon admission (E exo) and later in their hospitalization (L exo, average 19-day of hospitalization, *n* = 10), or non-COVID-19 donors (C exo, *n* = 8) in RPMI media at 37 °C for 16 h, followed by staining of cells with respective antibodies for flow cytometry. Exosome-depleted plasma of each donor group (E depl, L depl, and C depl) was used along with plasma exosomes. Isotype antibody controls and blank controls were performed in parallel. Data represent average ± SD, *n* = 10, one-way ANOVA; *p < 0.05. (**b**) Gating strategy for CD3^+^ lymphocytes, CD4^+^ T cells, and CD8^+^ T cells. Monocytes were gated for CD14 (Supplementary Fig. S2a).

We nest treated PBMC with COVID-19 plasma exosomes derived from patients obtained upon admission and again later in hospitalization, as well as non-COVID plasma exosomes to determine the immune response of PBMC to these exosomes. The average age of those patients was 58.3. We found that plasma exosomes from COVID-19 patients upon hospital admission significantly increased expression of IL-6, IL-8, and TNF-α in PBMCs gated for CD3^+^ lymphocytes, CD4^+^ T cells, CD8^+^ T cells, or CD14^+^ monocytes compared with plasma exosomes from the same patients later in their hospitalization or those from non-COVID donors (Fig. 2a,b, Supplementary Fig S2a). In contrast, COVID-19 plasma exosomes were unable to induce expression of IL-6 by CD8^+^ T cells relative to treatment with non-COVID-19 plasma exosomes. Importantly, exosome-depleted COVID-19 plasma failed to stimulate cytokine production in PBMCs, defining SARS-CoV-2-associated exosomes as agents with the capacity to elevate cytokine levels in COVID-19 patients.

We determined whether SARS-CoV-2-associated exosomes themselves initiated the response of PBMC to exclude potential confounding activation by contaminating viral particles using exosomes derived from Vero cells infected or not with a non-replicative SARS-CoV-2 construct. Thus, we treated PBMC with exosomes isolated from culture supernatants of SARS-CoV-2-ΔN/EGFP VERO E6 cells, followed by flow cytometry to assess cytokine expression. We found that exosomes derived from SARS-CoV-2-ΔN/EGFP VERO E6 cells significantly induced expression of IL-6 and TNF-α in CD14^+^ monocytes, and just IFNγ in CD8^+^ cells, with these experiments complicated by the inflammatory nature of Vero cell exosomes themselves (Supplementary Fig. 2a,b). These results additionally exclude a role for the N protein in the response to these exosomes.

### SARS-CoV-2-associated plasma exosomes differentially interact with immune cells

**Figure 3:**
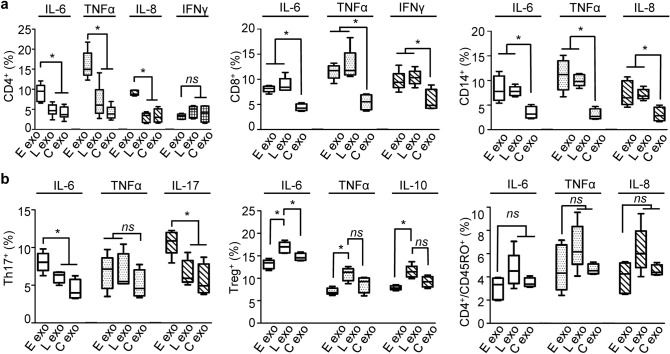
Cytokine production in subsets of immune cells in response to COVID-19 plasma exosomes. (**a**) MicroBeads sorted CD4^+^ T cells, CD8^+^ T cells, and CD14^+^ monocytes were treated with plasma exosomes (4 × 10^9^ ml^−1^) from the same patients (average age 52.1) early (E exo) in their admission and later (L exo) in their hospitalization (average 4 days) or non-COVID-19 donors (C exo) at 37 °C for 16 h. Cytokine production was quantified using flow cytometry gated for live CD4^+^, CD8^+^, CD14^+^ cells. (**b**) Cytokine production was quantified using MicroBeads selected CD4^+^ cells gating on Th17 T cells using PE-CF594-conjugated CD196 (CCR6) (Clone 11A9, BD Bio.) and on T_reg_ T cells using PE-conjugated CD25 (Clone 2A3, BD Bio.). CD4^+^ central memory T cells were separated using MicroBeads kit (Miltenyi Biotech) and gated for live cells. Isotype controls and no-antibody blank controls were used in parallel in flow cytometry. Data represent average ± SD; *n* = 10; *p < 0.05; *ns*, *p* > 0.05; one-way ANOVA equal variant.

To determine whether COVID-19 plasma exosomes directly regulated the immune response of a specific type of immune cells comprising PBMC, we separated CD4^+^ T cells, CD8^+^ T cells, and CD14^+^ monocytes from PBMCs using MACS MicroBeads (Miltenyi Biotec Inc.), followed by treatment with plasma exosomes and subsequent flow cytometry gating on live cells. We found for CD4^+^ T cells, COVID-19 plasma exosomes obtained from patients upon admission stimulated production of IL-6, IL-8, and TNF-α compared to plasma exosomes from non-COVID donors, with expression of IFNγ not being significantly affected (Fig. 3a, Supplementary Fig. 3a,b). CD4^+^ cells did not respond to exosomes obtained from these same COVID-19 patients later in their hospitalization. In contrast, for CD8 + T cells, plasma exosomes of the same patients obtained from early and later in their hospitalization increased IL-6, TNF-α, and IFNγ production compared with those from non-COVID donors. Similarly, COVID-19 plasma exosomes from patients in both their early and late hospitalization stimulated the expression of IL-6, IL-8, and TNF-α in CD14^+^ monocytes (Fig. 3a). Th-17 T cells are a subset of CD4^+^ T helper cells characterized by the production of IL-17, which may have evolved for host protection against microbes^33^. We found that plasma exosomes from early-stage patients stimulated IL-17 and IL-6 expression in CD4^+^ Th17 cells, while TNF-α production remained unchanged (Fig. 3b, Supplementary Fig. S3c). Regulatory T cells (_Tregs_) generate soluble factors, such as IL-10, to suppress activation, proliferation, and cytokine production of CD4^+^ T cells and CD8^+^ T cells^34^. COVID-19 plasma exosomes from patients later in their hospitalization induced the production of IL-6, TGFβ, and IL-10 compared with plasma exosomes from early-stage patients or non-COVID donors in Tregs (Fig. 3b, Supplementary Fig. S3c). In addition, COVID-19 plasma exosomes failed to affect cytokine production in CD4^+^/CD45RO^+^ central memory T cells that were selected using a CD4^+^ central memory T cell isolation kit (Miltenyi) (Fig. 3b, Supplementary Fig. S3c). Our findings suggested that select subpopulations of T cells, including Th-17 cells, T_reg_ cells, and CD4^+^ central memory T cells, were less responsive to COVID-19 plasma exosomes than CD4^+^ T cells, CD8^+^ T cells, and CD14^+^ monocytes.

### SARS-CoV-2 viral dsRNA contributes to immune responses to virus-associated exosomes

**Figure 4:**
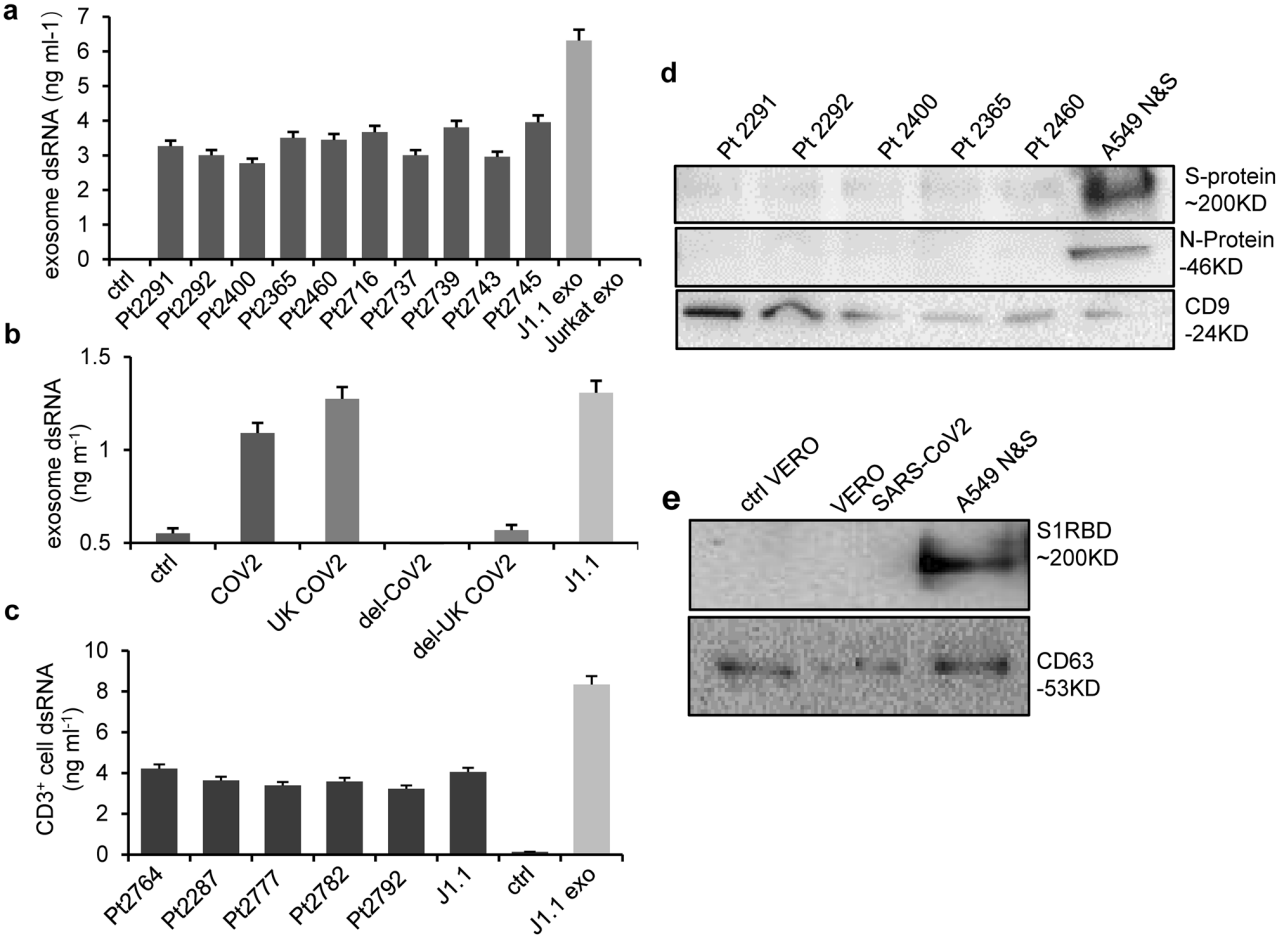
Viral dsRNA in COVID-19 plasma exosomes and recipient cells. (**a**) Quantification of dsRNA in COVID-19 plasma exosomes from patients (Pt2291 to Pt2745, average age 49.6) upon hospitalization. J1.1 exo, HIV + J1.1 T-cell exosomes; Jurkat exo, Jurkat T cell exosomes; ctrl, plasma exosomes derived from non-COVID donors. Data represent average ± SD, *n* = 3. The experiment was repeated 3 times independently. (**b**) dsRNA quantification in exosomes from control VERO E6 cells (ctrl), SARS-CoV-2-ΔN/EGFP VERO E6 cells (COV), and VERO E6 cells transfected with the UK variant of SARS-CoV-2-ΔN/EGFP (UK COV2). J1.1, J1.1 T-cell exosomes; del-COV2 and del-UK COV2, exosome-depleted cultural supernatants. Data represent average ± SD, *n* = 3. The experiment was repeated 3 times independently. (**c**) MicroBeads separated CD3^+^ lymphocytes from PBMC were treated with COVID-19 plasma exosomes from patients (Pt2764 to Pt2792, average age 61.2) or J1.1 HIV + T-cell exosomes (J1.1 exo) for 30 min, followed by dsRNA measurement in CD3^+^ lymphocytes. ctrl, non-COVID plasma exosomes. Data represent average ± SD of one experiment out of 3 independent repeats,* n* = 3. (**d**) Immunoblot of plasma exosome proteins (100 µg per lane) from COVID-19 patients (Pt2291to Pt2460, average age 42) and (**e**) A549-hsHA-Nflag cell exosomes overexpressing N and S proteins (A549 N&S). S-protein, SARS-CoV-2 S protein; N-protein, SARS-CoV-2 N protein; S1RBD, S protein blotted using the antibody to S1 receptor binding domain (RBD). CD9 (in **d**) and CD63 (in **e**) blots were used as exosome markers on the same blot.

A wide spectrum of viruses infects permissive cells producing viral polynucleotides that, when released from infected cells, subsequently induce innate immune responses via pattern recognition receptors (PRRs) that include the endosomal receptor TLR3^35–37^. We have reported that exosomes purified from the plasma of individuals infected with HIV and those isolated from culture supernatants of HIV-infected T cells contain viral double-stranded RNA (dsRNA), which induces the expression of proto-oncogenes and IFN-stimulated genes (ISGs) in cancer cells via TLR3^17^. SARS-CoV-2 infection and replication yield dsRNA intermediates, which are potentially involved in eliciting innate immune responses of respiratory tract-derived cells and cardiomyocytes^35^. To determine whether COVID-19 plasma exosomes contained dsRNA, we quantified dsRNA using a viral dsRNA detection system (PerkinElmer, Waltham, MA) based on a fluorescence resonance energy transfer (FRET) platform and a monoclonal dsRNA antibody^38^. We identified viral dsRNA in plasma exosomes from each COVID-19 patient we tested, which was not present in plasma exosomes from non-COVID donors or in exosomes from Jurkat T cells (Fig. 4a). Our positive control showed dsRNA in exosomes isolated from culture supernatants of HIV-infected J1.1 T cells, as expected^17^. The finding was validated by detection of dsRNA in exosomes isolated from culture supernatants of VERO E6 cells transfected with either SARS-CoV-2-ΔN/EGFP or the SARS-CoV-2-ΔN/EGFP UK variant. As expected, exosome-depleted supernatants did not contain dsRNA (Fig. 4b). To determine if COVID-19 plasma exosomes could transfer viral dsRNA into recipient cells, we incubated CD3^+^ lymphocytes with COVID-19 plasma exosomes, followed by washing and dsRNA quantification in these cells. We identified dsRNA in CD3^+^ lymphocytes incubated with COVID-19 plasma exosomes and HIV-infected J1.1 T-cell exosomes but not those treated with plasma exosomes from non-COVID donors (Fig. 4c). These results indicate that COVID-19 plasma exosomes are able to effectively transfer viral dsRNA cargoes to immune cells as we reported before^17^. To determine whether COVID-19 plasma exosomes contained viral S and/or N protein, we extracted total exosomal proteins (100 µg total protein for each loading) for immunoblotting using antibodies against the S and N proteins. We did not identify viral proteins in COVID-19 plasma exosomes that were positive for the exosome marker CD9, although exosomes from culture supernatants of A549 cells overexpressing S and N proteins contained both S and N proteins (Fig. 4d). Consistent with this observation, we did not detect the S protein in exosomes from culture supernatants of SARS-CoV-2-ΔN/EGFP VERO E6 cells, while the exosome marker CD63 protein was present (Fig. 4e). Although detection of SARS-CoV-2 protein(s) was reported in COVID-19 plasma exosomes immobilized in a chip platform^26^, our findings suggest that viral dsRNA may serve as a major cargo of COVID-19 plasma exosomes and, once delivered into recipient cells, may induce cellular responses.

### SARS-CoV-2 viral dsRNA contributes to immune responses to COVID-19 plasma exosomes

**Figure 5:**
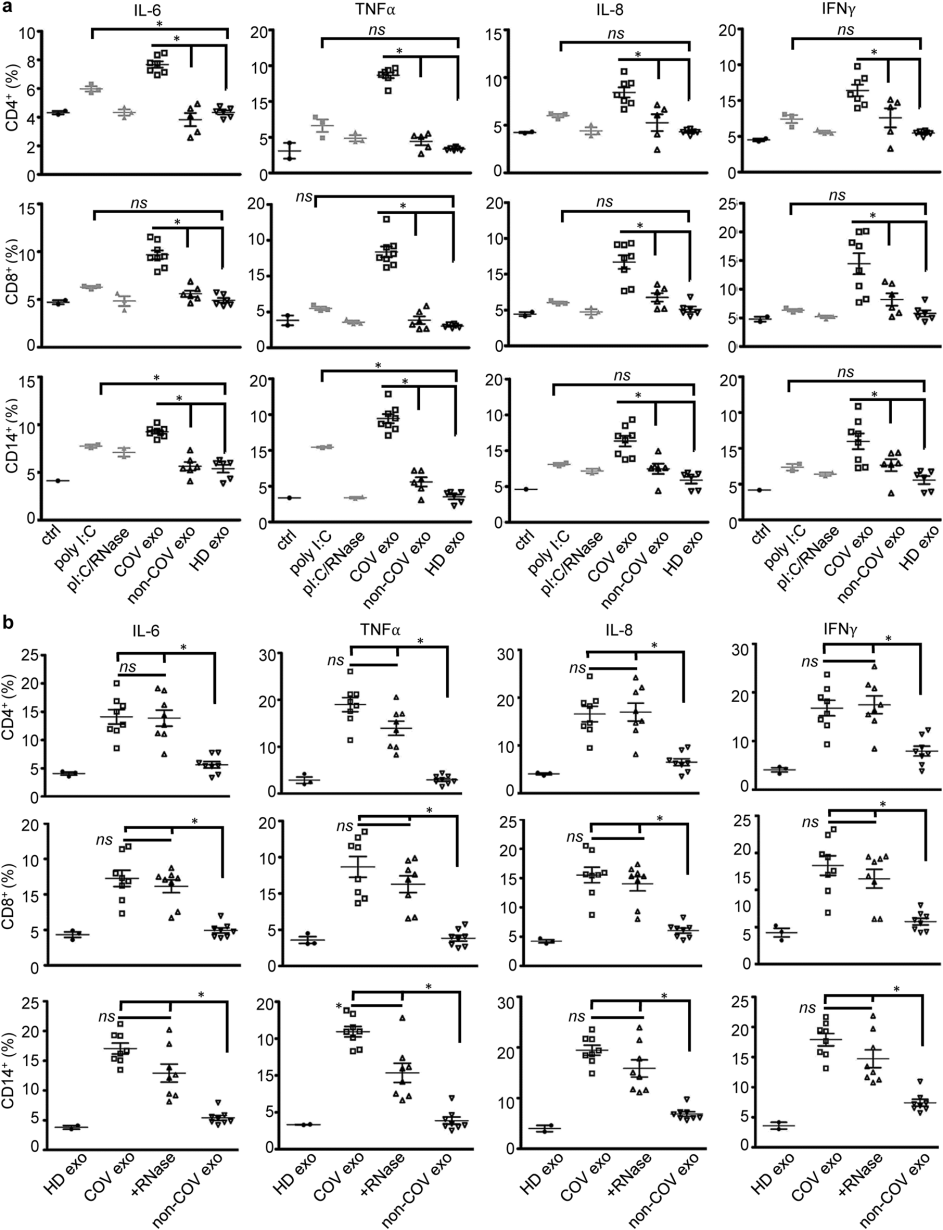
Production of cytokines in response to plasma exosomes and RNase. (**a**) CD4^+^ T cells, CD8^+^ T cells, and CD14^+^ monocytes were separated from PBMCs using MicroBeads, followed by treatment with plasma exosomes (4 × 10^9^ ml^−1^) from COVID-19 patients upon admission (COV exo, *n* = 8), non-COVID donors (non-COV exo, *n* = 6), healthy donors (HD exo, *n* = 5) as well as poly(I:C) (poly I:C, 5 µg ml^−1^) and poly(I:C) treated with RNase A (pI:C/RNase) in RPMI medium for 16 h at 37 °C. ctrl, medium only. Expression of cytokines was determined by flow cytometry gating on live cells. Data represent one experiment out of three repeats. Each flow cytometry assay was run in triplicate. Error bars, ± SD; *p < 0.05; *ns*, *p* > 0.05. One-way ANOVA. (**b**) COVID-19 plasma exosomes treated with an RNase (+ RNase, 50 µg ml^−1^ at 37 °C for 10 min) or remained untreated (COV exo) were used to stimulate PBMC for cytokine flow cytometry as in (**a**). Plasma exosomes from non-COVID (non-COV exo) and healthy donors (HD exo) were used as controls. Error bars, ± SD; *p < 0.05; *ns*, *p* > 0.05; one-way ANOVA. Graphs shown representative results from three biological repeats. Isotype antibody controls and blank controls were performed in parallel in flow cytometry.

TLR3 detects dsRNA derived from viral genomes released from damaged host cells, viral particles, and/or extracellular vesicles from infected cells^17,39,40^. Various cell types, including immune cells, epithelial cells, and endothelial cells, express TLR3 and respond to dsRNA through TLR3 signaling^41,42^. To determine whether COVID-19 plasma exosomes induce immune responses through viral dsRNA, we treated MicroBeads selected CD4^+^ T cells, CD8^+^ T cells, and CD14^+^ monocytes with plasma exosomes from COVID-19 patients and non-COVID donors as well as polyinosinic-polycytidylic acid (poly(I:C)), a synthetic analog of dsRNA and a potent activator of TLR3^43,44^, or poly(I:C) treated with an RNase. We found that COVID-19 plasma exosomes significantly induced expression of cytokines and chemokines in CD4^+^ and CD8^+^ T cells as well as in CD14^+^ monocytes compared with treatment with plasma exosomes derived from non-COVID-19 patients or healthy donors (Fig. 5a). This induction exceeded that induced poly(I:C), which moderately induced expression of IL-6 in CD4^+^ T cells and IL-6 and TNF-α in CD14^+^ monocytes compared with plasma exosomes obtained from healthy donors, and fully lacked an effect on expression of cytokines by CD8^+^ T cells (Fig. 5a). Our results suggested that exogenous dsRNA mimics were unable to recapitulate the function of exosomal viral dsRNA. We speculated that plasma exosomes may protect the viral RNA cargo from RNase degradation and effectively deliver it to recipient cells for signaling. Indeed, COVID-19 plasma exosomes treated with RNase were still induced expression of cytokines in lymphocytes and monocytes (Fig. 5b). These outcomes indicate that the viral dsRNA within COVID-19 plasma exosomes is positioned to play a unique role in the regulation of the immune responses of PBMC, and additionally may function to transmit pathogenic factors to non-permissive cells that, themselves, lack ACE2 or other SARS-CoV-2 ligation, processing, or internalization receptors.

**Figure 6:**
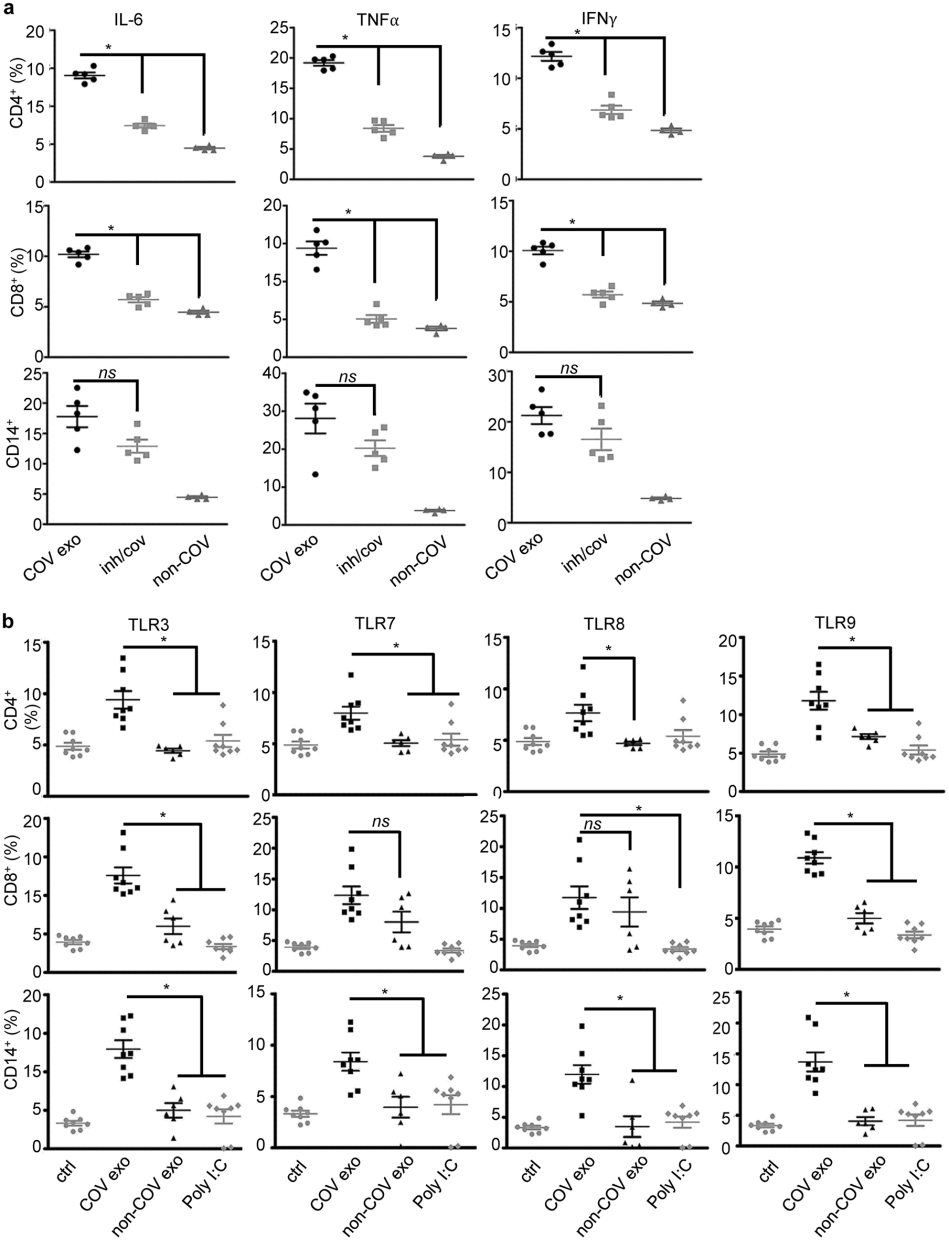
Cytokine production and TLRs in response to plasma exosomes. (**a**) PBMCs were treated with the TLR3 inhibitor (10 µM) for 30 min or remained untreated, followed by stimulation with plasma exosomes (4 × 10^9^ ml^−1^) for 16 h. Production of cytokines was determined by flow cytometry gated on CD4^+^, CD8^+^, and CD14^+^. COV exo, COVID-19 plasma exosome treatment; inh/cov, TLR3 inhibitor treated and COVID-19 plasma exosomes stimulated; non-COV, treatment with non-COVID plasma exosomes. *p < 0.05; *ns*, *p* > 0.05. ANOVA, equal variants. (**b**) CD4^+^ T cells, CD8^+^ T cells, and CD14^+^ monocytes were isolated from PBMC using MicroBeads, followed by treatment with plasma exosomes from COVID-19 patients upon admission (COV exo, 4 × 10^9^ ml^−1^), non-COVID donors (non-COV exo, 4 × 10^9^ ml^−1^), or poly(I:C) (5 µg ml^−1^) for 16 h at 37 °C. Expression of TLR3, TLR7, TLR8, and TLR9 was determined by flow cytometry gated on live cells. Isotype antibodies and no-antibody blanks were used in each flow cytometry assay. ctrl, medium only control. Error bars, ± SD; *p < 0.05; *ns*, *p* > 0.05; one-way ANOVA. Isotype antibody controls and blank controls were performed in parallel in flow cytometry.

We determined whether TLR3 mediated the immune response to COVID-19 plasma exosomes by treating PBMC with the dsRNA/TLR3 small molecular inhibitor, a competitive inhibitor of dsRNA binding to TLR3 with high affinity and specificity^17,45^, followed by stimulation with COVID-19 or non-COVID-19 control plasma exosomes and flow cytometry. Our results showed that the TLR3 inhibitor blocked the production of IL-6, TNF-α, and IFNγ in CD4^+^ T cells and CD8^+^ T cells, but in contrast the inhibitor failed to affect expression of cytokines in CD14^+^ monocytes (Fig. 6a). Thus, while TLR3 played a major role in cytokine production in T cells, monocyte pattern recognition receptors (PRRs) other than TLR3 must function in response to SARS-CoV-2-associated plasma exosomes. In addition, plasma exosomal proteins of COVID-19 patients may play a role in the immune response to SARS-CoV-2^25^. Given that some cytokines and chemokines induced by COVID-19 plasma exosomes, such as IL-6 and IL-8, are not typical signature cytokines or chemokines of T cells, we investigated how PRRs of T lymphocytes and monocytes responded to COVID-19 plasma exosomes. MicroBeads selected CD4^+^ T cells, CD8^+^ T cells, and CD14^+^ monocytes from PBMCs were treated with plasma exosomes from early-stage COVID-19 patients and non-COVID donors for 16 h at 37 °C, followed by flow cytometry for expression of TLR3, TLR7, TLR8, and TLR9 gating on live cells. We found that COVID-19 plasma exosomes significantly induced the expression of TLR3 and TLR9 in all subsets of immune cells tested, while COVID-19 plasma exosomes were unable to induce TLR7 expression in CD8^+^ T cells (Fig. 6b). The expression of TLR8 in CD8^+^ T cells was not significantly different between COVID-19 and non-COVID-19 plasma exosome treatments. Poly(I:C) failed to stimulate expression of any of these TLRs in these various PBMCs. Our findings suggest that COVID-19 exosomes derived from SARS-CoV-2-infected cells sensitize innate and adaptive immune cells to exosomal viral cargoes enabling proinflammatory cytokine/chemokine responses. These events may contribute to the severity and delayed recovery of COVID-19.

## Discussion

The goal of this study was to reveal the role of plasma exosomes released by SARS-CoV-2-infected cells in the regulation of immune responses in COVID-19 patients, including peripheral immune cells that are nonpermissive for SARS-CoV-2 infection. We purified exosomes and obtained exosome-depleted plasma simultaneously from plasma specimens collected from hospitalized COVID-19 patients upon admission and up to 86 days after their hospital admission. We found that over 90% of COVID-19 plasma exosome samples contained SARS-CoV-2 RNA using the method designated for detection of viral RNA in saliva and nasal swab specimens. SARS-CoV-2-ΔN/EGFP VERO E6 cells do not release virions; however, exosomes isolated from culture supernatants of VERO E6 cells contain viral RNA. This is consistent with observations from a previous report, in which critical and noncritical COVID-19 patients contained SARS-CoV-2 RNA in their plasma exosomes, although the RNA copy numbers between the two patient groups were similar^25^. Others also reported the presence of SARS-CoV-2 RNA in plasma exosomes of COVID-19 patients in their acute phase of infection^25,46^. Moreover, SARS-CoV-2 RNA fragments have now been identified in platelet-derived extracellular vesicles^47^. Although SARS-CoV-2 RNA is present in the exosomal cargo, whether the virus might employ the structural features of the viral RNA for signaling or delivery of the viral genome for infection remains to be further investigated. Interestingly, neither the S nor N protein of SARS-CoV-2 was detected in exosomes derived from COVID-19 plasma or culture supernatants of SARS-CoV-2-ΔN/EGFP VERO E6 cells by immunoblotting in our studies. Similarly, proteomic approaches have been unable to detect viral proteins in COVID-19 plasma exosomes^25,46^. In exosomes isolated from A549 cells that overexpressed the S and N proteins, however, we identified both proteins using immunoblotting. This is consistent with observations reported by Troyer et al., in which S and N proteins are present in exosomes isolated from the culture media of cells overexpressing viral proteins^48^. This raises the possibility that exosomes isolated from cells that overexpress SARS-CoV-2 proteins may not represent exosomes purified from COVID-19 patient blood specimens or other body fluids. A recent report detected SARS-CoV-2 protein(s) in immunoprecipitated COVID-19 plasma exosomes or exosomes immobilized in a chip platform^26^. This report also presented transmission electron microscopy data identifying SARS-CoV-2 S protein in COVID-19 plasma exosomes using gold-conjugated antibody to the RBD of S protein^26^, suggesting the need for further investigation on the matter. Our findings indicate that plasma exosomes are able to protect their cargo of dsRNA from RNase degradation and then effectively transfer the dsRNA to recipient cells for signaling. Thus, COVID-19 plasma exosomes may play an important role in eliciting immune responses of peripheral immune cells.

Our findings strongly support COVID-19 plasma exosomes as a risk factor able to intensify COVID-19 through stimulation of the proinflammatory response of peripheral blood immune cells. We found that plasma exosomes from COVID-19 patients significantly induced the production of the same set of cytokines and chemokines that contribute to the severity of COVID-19^49–53^, including IL-6, IL-8, TNF-α, IFNγ, CCL1, and GDF-15, in PBMCs compared with those from non-COVID donors. Exosomes isolated from culture supernatants of SARS-CoV-2-ΔN/EGFP VERO E6 cells similarly induced cytokine production in CD14^+^ monocytes. It has been reported that hospitalized SARS-CoV-2-positive patients produce significantly higher levels of serum IL-6, IL-8, TNF-α, and IL-1β; each of these cytokines is independently predictive of overall survival^49^. Elevated serum levels of IL-6, IL-8, IL-1β, and TNF-α correlate with moderate and severe COVID-19, while IL-12p70 and IL-2 serum expression is associated with asymptomatic and mild diseases^50,51^. Serum levels of IL-1β, IL-6, and IL-8, together with several complement components, positively correlate with critical COVID-19 in-hospital deaths^52^. Thus, serum IL-6, IL-8, IL-β, and TNF-α are independent and significant predictors of disease severity and death in COVID-19^49–53^. In addition to these cytokines, elevated serum GDF-15, which we found to be increased by COVID-19 plasma exosomes, was associated with most hospitalized COVID-19 patients and SARS-CoV-2 viremia, hypoxemia, and worse outcome^54^. In addition, our data suggest that the baseline inflammatory profile of patients, such as senescence and obesity, may not have much impact on the immune response by PBMC as these cells were isolated from healthy donors. Increased production of TNFα by CD4^+^ T cells and CD8^+^ T cells in response to COVID-19 plasma exosomes may contribute to differentiation of Th17 T cells. Our findings are in line with these clinical observations and indicate that SARS-CoV-2-associated plasma exosomes have the potential to contribute to excessive cytokine/chemokine responses in COVID-19 patients.

SARS-CoV-2 primarily infects host cells by binding to the cell surface receptor ACE2 followed by serine protease, such as TMPRSS2, processing or through the endocytic pathway within the endosomal–lysosomal compartments^55^. However, lymphocytes and monocytes do not express ACE2 and are not primary target cells for infection; thus, it remains unclear how the virus can interact with immune cells to modulate their responses. Exosomes are assembled as lipid bilayer vesicles with surface proteins; they can be taken up by direct fusion with the plasma membrane or by directly interacting with surface receptors of recipient cells^56^ and so need no high affinity receptors. We have reported that exosomes from latently HIV-infected T cells interact with the epidermal growth factor receptor (EGFR) of oral and lung cancer cells for cellular entry and subsequently stimulate downstream EGFR signaling without causing EGFR activation^17,18^. In this report, we demonstrate that incubation of lymphocytes with COVID-19 plasma exosomes leads to the accumulation of viral dsRNA in recipient lymphocytes. Our findings thus indicate that SARS-CoV-2-associated exosomes can act as pathogenic carriers to deliver and release viral cargoes of infected cells into immune cells, even those lacking the high affinity ACE2 SARS-CoV-2 receptor, potently stimulating expression of proinflammatory cytokines. This raises the prospect that COVID-19 plasma exosomes may have a broad spectrum of pathogenic impacts on diverse host cell types and contribute to the variety of disorders associated with SARS-CoV-2 infection.

Upon SARS-CoV-2 infection and entry, viral genome replication produces viral dsRNA intermediates that can be identified as early as 16 h after infection^57^. Since cells release exosomes consistently under physiological and pathological conditions, our findings provide strong evidence that SARS-CoV-2 transcribed dsRNA quickly circulates in patients at the time of admission to our hospital system protected within exosomes. These dsRNA-containing COVID-19 exosomes induce TLR-stimulated cytokine synthesis by some, although not all, immune cells. The release of viral exosomal cargoes inside immune cells leads to the interaction of cargo components with TLRs situated in the endosome, including TLR3, TLR7, TLR8, and TLR9^58,59^. TLR3 senses dsRNAs derived from viruses, small interfering RNAs, and poly(I:C) to induce the expression of IL-6, IL-8, TNF-α and other proinflammatory cytokines and chemokines^39,60,61^. Both TLR7 and TLR8 recognize single-stranded RNA (ssRNA) derived from viruses such as HIV, influenza, and HCV^62,63^. TLR9 is activated by unmethylated CpG sequences in DNA molecules, resulting in the production of inflammatory cytokines such as IL-6 and TNF-α in immune cells such as monocytes, dendritic cells (DCs), macrophages, and B cells that express TLR9^39,64^. In this report, we demonstrate that COVID-19 plasma exosomes stimulate protein expression of TLR3 as well as TLR7 and TLR9 in CD4^+^ T cells, CD8^+^ T cells, and CD14^+^ monocytes compared with cells that remained untreated, treated with plasma exosomes from non-COVID-19 or healthy donors, or poly(I:C). Given the involvement of endosomal TLRs in the pathogenesis of infection and autoimmune diseases, our results suggest that COVID-19 exosomes sensitize immune cells to pathogenic viral exosomal cargoes to produce proinflammatory cytokines and chemokines, including those that are not signature cytokines produced by T cells. Thus, our findings indicate that endosomal TLRs may serve as therapeutic targets for immune complications of COVID-19. In addition, COVID-19 plasma exosomes may present an acute pathogenic insult that contributes to dysfunctional immune response of patients. Additionally, viral RNA-positive plasma exosomes in patients, which extended to later the course of their hospitalization, have the potential to contribute to long-haul syndromes COVID-19. It has been reported that the proteomic components of plasma exosomes from COVID-19 patients and recovered patients are significantly distinct from those isolated from the plasma of healthy donors. These exosomal proteins include those involved in lipid metabolism and cellular responses to stress oxygen-containing compounds^46^. Although these exosomes are useful in monitoring outcomes of recovered COVID-19 patients for coagulation, inflammation, and organ function, we propose that proteins differentially packaged in COVID-19 plasma exosomes have an extended potential to directly impact production of cytokines and chemokines, even in cells and tissues not infected with SARS-CoV-2. The impact of exosomes released by infected cells on comorbidities of COVID-19 patients requires further investigation.

In conclusion, we demonstrate that plasma of COVID-19 patients contains exosomes derived from SARS-CoV-2 infected cells that usher viral cargoes throughout the circulation. This includes viral dsRNA, whose entry into peripheral immune cells, regulates expression of proinflammatory cytokines through TLRs, thereby contributing to a cytokine storm and disease progression. This novel mechanism is the first evidence of COVID-19 plasma exosomes orchestrating crosstalk of infected cells, primarily epithelial cells in the respiratory tract, with immune cells and other types of non-permissive cells that can contribute to COVID-19-associated disorders. This work also elucidates the relevance of targeting TLRs as a potential therapeutic strategy for this disease.

## Methods

### Ethical statement

Institutional Review Boards (IRB) of the University Hospitals Cleveland Medical Center approved the study (STUDY20201187) according to the 2003 Helsinki Declaration. Written informed consent was obtained from all study participants. Study participant’s names and other Health Insurance Portability and Accountability Act (HIPAA) identifiers were removed from data and samples obtained from the IRB-approved University Hospitals COVID-19 and Coronavirus Biorepository (STUDY20200517) and from all sections of the manuscript, including supplementary information.

### Cell cultures

The J1.1 cell line was obtained from the NIH AIDS Reagent Program. Jurkat cells were purchased from American Type Culture Collection (TIB-152, ATCC, Manassas, VA). These cells were maintained in RPMI 1640 medium (HyClone Lab., Inc., Logan, UT) supplemented with 10% exosome-depleted FBS, which was prepared by ultracentrifugation of FBS (ThermoFisher Scientific, Waltham, MA) at 100,000×*g* for 16 h at 4 °C, followed by collection of the supernatants without disturbing the pellet. VERO E6 cells, SARS-CoV-2-ΔN/EGFP BAC-transfected VERO E6 cells, and UK variant of SARS-CoV-2-ΔN/EGFP BAC-transfected VERO E6 cells were maintained in DMEM (HyClone Lab). A549-hsHA-Nflag cells were maintained in base medium F12/K (Gibco, Waltham, MA) supplemented with puromycin (20 μg ml^−1^) and hygromycin B (300 μg ml^−1^). Peripheral blood mononuclear cells (PBMCs) were isolated from healthy volunteers’ peripheral blood by a standard preparation method. Briefly, 20 ml of whole blood in 50 ml polystyrene tubes was diluted 1:1 with RPMI (HyClone Lab), underlaid with 10 ml of Ficoll-Paque Plus (cat# 17-1440-03, GE Healthcare, Chicago, IL). The tubes were then centrifuged at 1600 rpm for 20 min at room temperature. The white blood cell layer on top of the Ficoll, containing lymphocytes and monocytes, was transferred into new 50 ml tubes and washed with PBS.

### Exosome preparation and quantification

All COVID-19 plasma specimens were heated at 57 °C for 30 min to inactivate SARS-CoV-2 while keeping exosomes intact and functional before use^14,29,30^. Plasma samples from matched non-covid hospitalized donors were heat-treated in parallel. Plasma exosomes were prepared by the differential ultracentrifugation method as we reported previously^17^. Briefly, 200–250 μl of plasma was centrifuged at 400×*g* for 15 min to remove cell debris. The same volume of PBS (Lonza, Portsmouth, NH) was added to the supernatant, followed by centrifugation at 11,000×*g* for 10 min to remove any possible microvesicles and apoptotic bodies. Plasma exosomes in the supernatants were pelleted by ultracentrifugation at 100,000×*g* for 90 min at 4 °C (Optima™ Max-XP, 50.2Ti rotor, Beckman Coulter Inc., Brea, CA) and suspended in PBS. To isolate exosomes from cell culture supernatants, cell culture media were centrifuged at 400×*g* for 5 min to remove cells, followed by centrifugation at 11,000×*g* for 10 min to remove any possible apoptotic bodies and large cell debris. Exosomes were precipitated at 100,000×*g* for 90 min at 4 °C (50.2Ti rotor, Beckman Coulter) and suspended in PBS.

Isolated exosomes were quantified using the acetylcholinesterase (AChE) assay system (System Biosci. Inc (SBI), Palo Alto, CA) following the manufacturer’s instructions. Briefly, 20 μl suspended exosomes were mixed with 80 μl of Exosome Lysis Buffer to extract exosome proteins. After centrifugation at 1500×*g* to remove debris, the supernatants were mixed with the same volume of AChE reaction buffer on a microtiter plate, incubated at room temperature for 20 min and then read on a microplate reader at 405 nm. Exosomes were quantified as numbers of exosomes per ml. The yield of plasma exosomes was typically approximately 2 × 10^8^ exosomes per 10 µl. Nanoparticle tracking analysis was performed using ZetaView (Particle Matrix GmbH, Inning am Ammersee, Germany) following the manufacture's instructions.

### Flow cytometry and antibodies

CD3^+^, CD4^+^, CD8^+^, and CD14^+^ cells were isolated from PBMCs using cell-type specific MicroBeads (MACS, Miltenyi Biotec, North Rhine-Westphalia, Germany) following the manufacturer’s instructions. Briefly, 1 × 10^8^ PBMCs suspended in 800 µl of buffer were mixed with 200 µl of MACS MicroBeads and incubated for 15 min at 4–8 °C. The LS column was placed in the magnetic field of the MACS Separator. After washing with 3 ml of buffer, the cell suspension was applied on top of the column. The negative cells passed through. After rinsing with 3 ml of buffer (three times), the column was removed from the separator and then pipetted with 5 ml of buffer to flush out the positive. Purified CD3^+^, CD4^+^, CD8^+^, or CD14^+^ cells were cultured in 24-well plates individually and treated with plasma exosomes (4 × 10^9^ ml^−1^) for 16 h in a cell culture incubator at 37 °C. Cells were harvested and washed 3 times with PBS, resuspended in 100 µl of 0.5% PBS and permeabilized using an Intracellular Fixation & Permeabilization Buffer Set (eBioscience, Inc., San Diego, CA). Whereas polyinosinic-polycytidylic acid (poly(I:C)) was applied in treatment, poly(I:C) was added to cell cultures to 5 µg ml^−1^. Poly(I:C) treated with RNase A (ThermoFisher Sci., Waltham, MA) at 37 °C for 30 min was used as a negative control for poly(I:C) treatment. For flow cytometry, cells were stained using monoclonal antibodies: PE-conjugated IL-6 (Clone MQ2-13A5, BD Biosciences, Franklin Lakes, NJ), APC-conjugated TNF-α (Clone MAb11, BD Biosciences), PE-CF594-conjugated IL-8 (Clone G265-8, BD Biosciences), PE-CF594-conjugated IL-10 (Clone JES3-19F1, BD Biosciences), APC-R700-conjugated IL-17 (Clone N49-653, BD Biosciences), PerCP-Cy5.5-conjugated IFNγ (Clone B27, BD Biosciences), PerCP-Cy5.5-conjugated TGFβ (Clone TW4-9E7, BD Biosciences), PE-conjugated TLR3 (Clone TLR-104, Biolegend, San Diego, CA), PerCP-conjugated TLR7 (Clone 533707, R&D systems), FITC anti-human CD288 (TLR8) antibody (Clone 16018A, Biolegend), and APC-conjugated TLR9 (Clone S16D, Biolegend). Isotype control antibodies were APC mouse IgG1 (Clone MOPC-21), PE-CF594 mouse IgG2b (Clone 27–35), APC-R700 mouse IgG1 (Clone X40), PerCP-Cy5.5 mouse IgG1 (Clone X40), PE mouse IgG2a (Clone MOPC-173), and PerCP mouse IgG2a (Clone X39) from BD Biosciences. The dsRNA/TLR3 complex inhibitor was purchased from Millipore Sigma (cat# 614310, Burlington, MA). CD4^+^ central memory T cells were isolated from CD4^+^ T cells using the human CD4^+^ Central Memory T Cell Isolation Kit (Miltenyi Biotec). Flow cytometry data were analyzed using a FACSAria Flow Cytometer (BD Biosciences), and FACS data were analyzed with CytExpert (Beckman Coulter Inc.). For all flow cytometry experiments, isotype antibody and blank controls were used to eliminate backgrounds.

### Immunoblotting

Total exosome proteins were extracted and purified using the Total Exosome RNA & Protein Isolation Kit (ThermoFisher) following the manufacturer’s instructions with a protease inhibitor cocktail (ThermoFisher). Protein lysates were separated by SDS-PAGE and then transferred onto polyvinylidene fluoride membranes (PVDF, MilliporeSigma Inc.) for immunoblotting. The membranes were blocked in 5% milk for 1 h at room temperature, followed by incubation with antibodies against CD9 (1:1000; TS9, Thermo Fisher), CD63 (1:1000; NKI/C3, Novus Biologicals, Littleton, CO), SARS-COV-2-S1RBD (1:1000; 1F10-D4-B1, RayBiotech, Peachtree Corners, GA), and SARS-COV-2 N-Protein (1:1000; 5F7-A3, RayBiotech) overnight at 4 °C. The membranes were then washed and incubated with appropriate HRP-conjugated secondary antibodies for 1 h at room temperature. Protein detection was performed by chemiluminescence using an ECL kit (ThermoFisher).

### Qualitative detection of SARS-CoV-2 nucleic acids

SARS-CoV-2 nucleic acids in COVID-19 plasma exosome samples were qualitatively detected using the COVID-19 1-Step High Throughput PCR Kit (RayBiotech) following the manufacturer’s instructions. Briefly, COVID-19 plasma exosomes (5 μl) or exosome-depleted plasma (5 μl) was directly mixed with PCR Enzyme (1 μl) and Mix PCR Reaction Solution (14 μl). The positive control reaction and negative control reaction were set up accordingly. The total reaction volume was 20 µl. SYBR Green fluorescence was detected by the Real-time PCR System (SteponePlus, ABI, Waltham, MA). The final fluorescence value (F) was divided by the cycle 10 fluorescence value (10). A ratio value (F/10 ratio) > 0.85 was considered positive according to the manufacturer’s instructions on human samples.

### SARS-CoV-2 nucleic acid quantification

Total RNA was extracted from COVID-19 plasma exosomes, SARS-COV-2/SARS-COV-2 alpha VERO E6 cell exosomes, and A549 N&S exosomes using the High Pure RNA Isolation Kit (Roche Life Sci., Basel, Switzerland). SARS-CoV-2 S and N genes were detected with a SARS-CoV-2 SYBR Green RT-qPCR Quantification Kit (ScienCell, Inc., Carlsbad, CA) following the manufacturer’s instructions. Briefly, COVID-19 plasma exosome RNA was reverse-transcribed to cDNA (2 μl) and mixed with the SARS-CoV-2 S gene primer set, SARS-CoV-2 N gene primer set, and human ACTB gene primer set. The human ACTB gene primer set targets the human β-actin (ACTB) housekeeping gene, which serves as a normalization control for cell number quantification. qPCR was performed in a 20 µl volume, and SYBR Green fluorescence was detected by the Real-time PCR System (SteponePlus, ABI). Ct values less than 35 were considered positive according to the manufacturer’s instructions on using human samples.

### SARS-CoV-2 viral dsRNA detection

Purified PBMCs were cultured in 24-well plates and treated with COVID-19 plasma exosomes for 30 min. PBMCs treated with exosomes isolated from culture supernatants of HIV + J1.1 T cells were used as positive controls. After treatment, the cells were harvested and washed 3 times with PBS for dsRNA detection using a viral double-stranded RNA detection kit (PerkinElmer, Inc., Waltham, MA) following the manufacturer’s instructions. Briefly, 10 µl of PBMCs was dispensed into each sample well, followed by the addition of 5 µl of dsRNA d2 antibody working solution and 5 µl of dsRNA Eu Cryptate antibody working solution. After incubation of the plate at 4 °C overnight, the plate was read on the HTRF compatible reader Synergy H1 (BioTek Instruments, Winooski, VT).

### Antibody array assays

Purified PBMCs incubated in 24-well plates were treated with plasma exosomes (4 × 10^9^ ml^−1^) for 16 h. Culture supernatants were collected, and the production of cytokines in PBMCs was detected using the G-Series Human Cytokine Antibody Array 1000 (G7 and G8 slides) (RayBiotech) following the manufacturer’s instructions. Briefly, glass slides were blocked with 100 µl of sample diluent, incubated at room temperature for 30 min and then incubated with 100 µl of culture supernatant in each well overnight at 4 °C. After washing with 150 µl of 1 × Wash Buffer I (three times, 5 min each) and 150 µl of 1 × Wash Buffer II (two times, 5 min each) to remove unbound cytokines, glass slides were incubated individually with 80 µl of the detection antibody cocktail at room temperature for 2 h. After washing, 80 µl of Cy3 equivalent dye-conjugated streptavidin was added to each well and incubated in the dark for 1 h. After removing the slide from the gasket and washing extensively, signals were detected by the Axon GenePix laser scanner equipped with a Cy3 wavelength (green channel). Data extraction was performed using the GAL file along with the microarray analysis software, and data analysis was performed using array-specific RayBio Analysis Tools (RayBiotech).

### Statistics

Each experiment was repeated at least three times. The results of the treatments were compared with those of the respective controls. Data are presented as the mean ± SD. Flow cytometry data were subjected to one-way ANOVA. Statistical significance was considered at *p* < 0.05. Data analyses were performed, and graphs were generated using Prism (GraphPad Software, La Jolla, CA) and Excel 2016 (Microsoft, Redmond, WA).

## Supplementary Information

Supplementary Information.

## Data Availability

All data that support the findings of this study are available from the corresponding author upon request.
