# Supplementary material for: COVID-19 plasma exosomes promote proinflammatory immune responses in peripheral blood mononuclear cells
Source: Sci Rep. 2022 Dec 16;12:21779. doi: 10.1038/s41598-022-26457-8 (PMC9756928; doi:10.1038/s41598-022-26457-8)
Supplement: Supplementary file 1 — Supplementary Information. [file 41598_2022_26457_MOESM1_ESM.pdf]

Supplemental information

COVID-19 plasma exosomes promote proinflammatory immune responses in peripheral blood mononuclear cells

Lechuang Chen[1], Rui Chen[2], Min Yao[3], Zhimin Feng[1], Guoxiang Yuan[1], Fengchun Ye[4], Kien Nguyen[4], Jonathan Karn[4], Grace A. McComsey[5], Thomas M. McIntyre[2], Ge Jin[1]

[1]Department of Medicine, Case Western Reserve University School of Medicine, the MetroHealth Medical Center, Cleveland, Ohio 44109, USA.

[2]Department of Cardiovascular and Metabolic Sciences, Cleveland Clinic Lerner Research Institute, Cleveland, Ohio 44195, USA.

[3]Department of Radiation Oncology, Penn State Cancer Institute, the Pennsylvania State University College of Medicine, Hershey, Pennsylvania 17033, USA.

[4]Department of Molecular Biology and Microbiology, Case Western Reserve University School of Medicine, Cleveland, Ohio 44106, USA.

[5]Department of Pediatrics and Medicine, Case Western Reserve University School of Medicine, University Hospitals Cleveland Medical Center, Cleveland, Ohio 44106, USA.

Supplementary Table 1. *Patient parameters*

	Basis	Percentage
**Total**	50	100
**Gender**		
male	23	46.0
female	27	54.0
**Age**		
under 30	4	8.0
31-49	12	24.0
50-79	28	56.0
over 80	6	12.0
**Social History**		
Current Smoker	6	12.0
Former Smoker	7	14.0
Alcohol Use Disorder	2	4.0
Substance Abuse Disorder	2	4.0
**Baseline syptoms**[1]		
Obesity	4	36.0
CAD	5	45.0
Hypertension	7	64.0
**Days of plasma collection between admission and later in hospitalization**		
1-7 days	36	72.0
over 8 days	14	28.0
**White Blood Cell Count**		
upon admission low	15	26.0
later in hospitalization low	5	10.0
**Lymphocyte Count**		
upon admission Low	18	36.0
later in hospitalization Low	4	8.0
**C Reactive Protein high**[2]	46	92.0
**Interleukin 6**[3]		
Abnormal High (pg/ml)	8	16.0

[1]data available for 11 patients, CAD, coronary artery disease; [2]abnormal (≥ 5 μml^{-1}) any day during hospitalization; [3]patient range 8.3-255.1, reference rage ≤ 2.0;

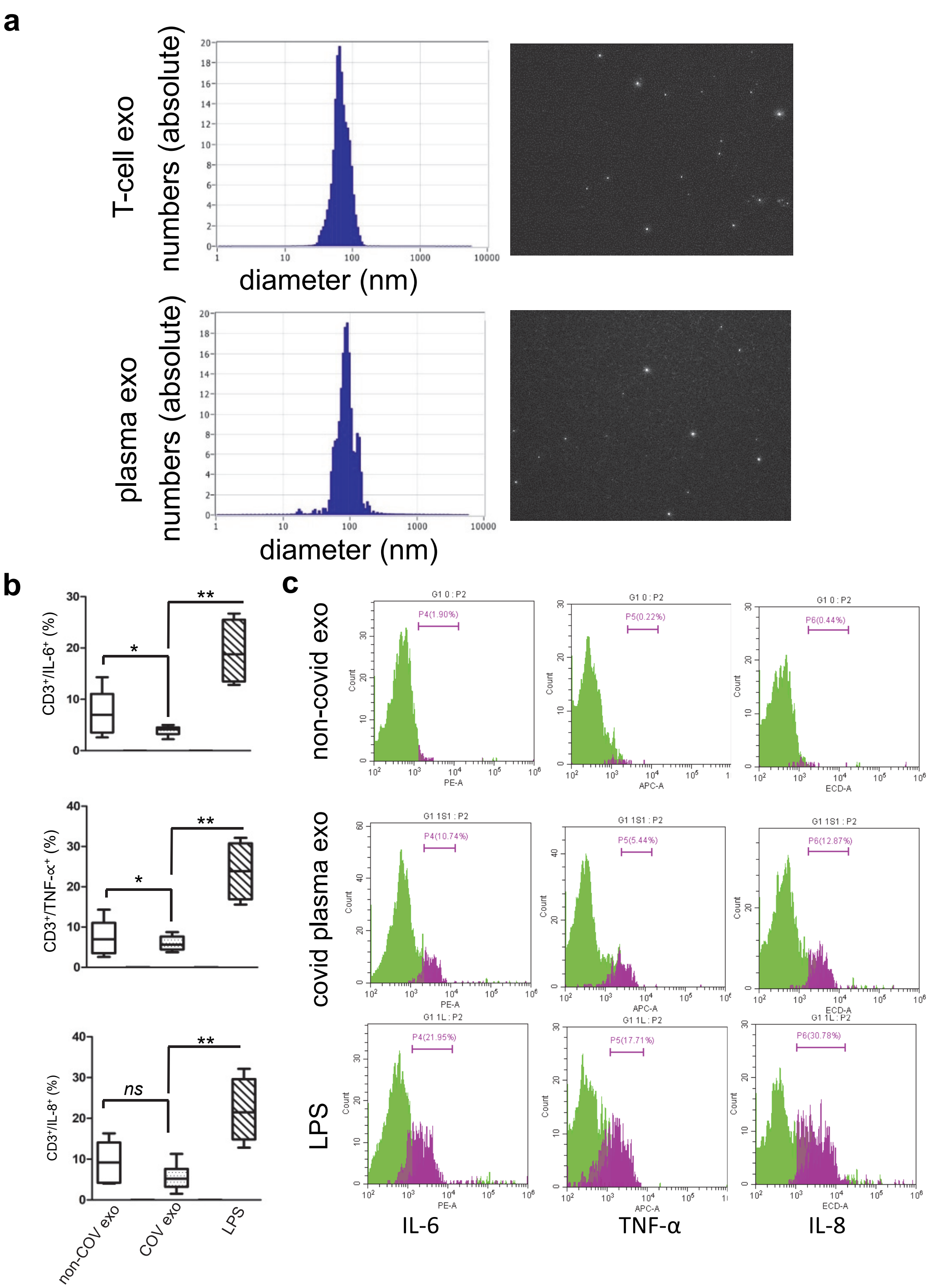

Figure S1. Nanopartical tracking analysis of exosomes and immune responses of CD3+ lymphocytes to COVID-19 plasma exosomes. (**a**) Size distribution of exosomes from culture supernatants of T cells (T-cell exo) and plasma of healthy donors (plasma exo) determined by the nanoparticle tracking analyzer ZetaView. Screenshots of particles in right panels. (**b**) PBMC were treated with plasma exosomes ($2x10^9$ m^{-1}) from COVID-19 patients (covid plasma exo) and non-COVID-19 donors (non-covid exo) as well as LPS (10 μg ml^{-1}) for 16 h at 37°C, followed by flow cytometry to quantify IL-6, IL-8, and TNF-α in PBMC gated on $CD3^+$ lymphocytes as described in Fig. 2b. Percentages of cytokine positive cells were labeled. Isotype antibody control and blank control assays were performed in parallel. Data represent average ± SD, n=10, one-way ANOVA equal variant; *, $p< 0.05$; **, $p<0.01$. (**c**) Histograms of b.

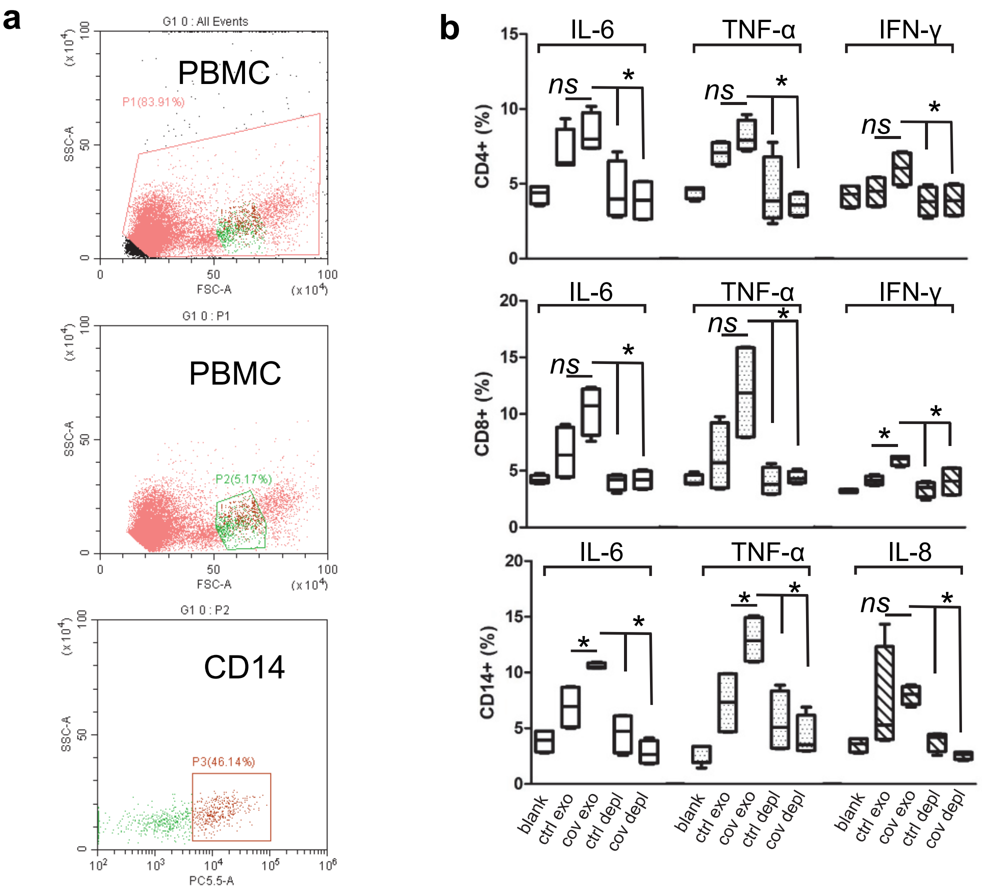

Figure S2. (**a**) Gating strategy for CD14⁺ monocytes in PBMC. (**b**) Expression of IL-6, IL-8, TNF-α, and IFNγ in PBMC treated with exosomes (4×10^{9} ml^{-1}) from culture supernatants of control VERO E6 cells (ctrl exo), SARS-CoV-2-ΔN/EGFP cells (cov exo), remained untreated (blan), and exosome-depleted culture supernatants from control (ctrl depl) and SARS-CoV-2-ΔN/EGFP cells (cov depl) for 16 h at 37 ℃, followed by flow cytometry as in Figure 2. Data represent average ±SD; *n*=5; * $p < 0.05$; *ns*, $p > 0.05$; one-way ANOVA. Isotype antibody controls and blank controls were performed in parallel in flow cytometry.

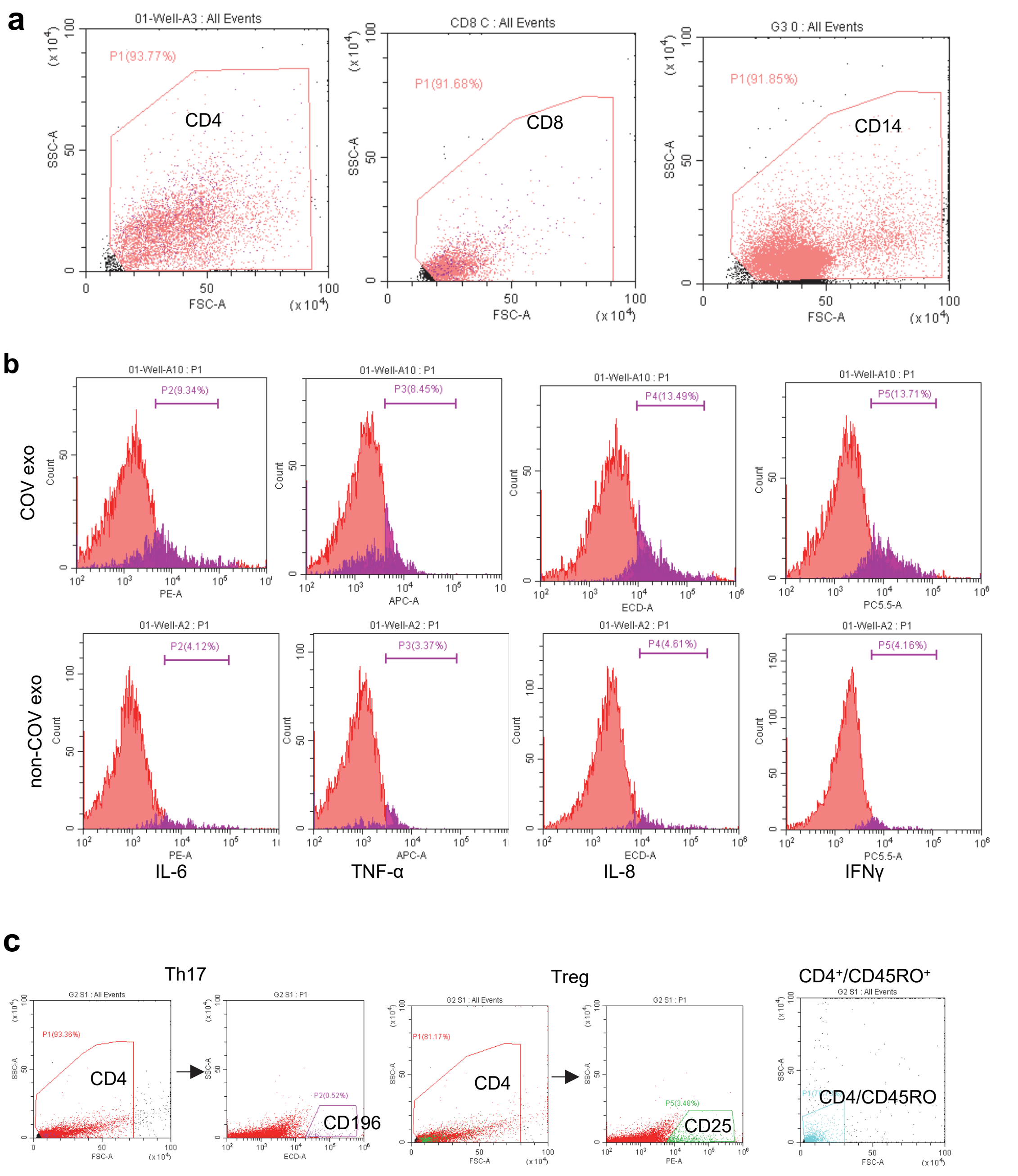

Figure S3. Response of subsets of immune cells to plasma exosomes. (**a**) $CD4^+$ T cells, $CD8^+$ T cells, and $CD14^+$ monocytes were isolated from PBMCs using MicroBeads and gated on live cells. (**b**) Representative flow cytometry graphs of IL-6, TNF-α, IL-8, and IFNγ production in MicroBeads selected $CD4^+$ T cells in response to COVID-19 plasma exosomes (COV exo) from patients early in admission and non-COVID-19 plasma exosomes (non-COV exo). Percentage of cytokine expressing cells were presented. (**c**) MicroBeads selected $CD4^+$ T cells were gated on live cells and $CD196^+$ (CCR6) for Th17 T cells, $CD4^+$ and $CD25^+$ for Tregs. $CD4^+$ central memory T cells were selected using the Human Central Memory T Cell Isolation kit (Miltenyi Inc.) and gated on live cells. Isotype antibody controls and blank controls were performed in parallel.
